# Phytochemical analysis of hydroethanolic extract of *Turnera diffusa* Willd and evaluation of its effects on astrocyte cell death

**DOI:** 10.1590/S1679-45082016AO3386

**Published:** 2016

**Authors:** Andréia Gomes Bezerra, Giuseppina Negri, Joaquim Maurício Duarte-Almeida, Soraya Soubhi Smaili, Elisaldo Araújo Carlini

**Affiliations:** 1 Universidade Federal de São Paulo, São Paulo, SP, Brazil.; 2 Universidade Federal de São João del-Rei, Divinópolis, MG, Brazil.

**Keywords:** Cognition/drug effects, Plant extracts/toxicity, Protective agents/toxicity, Antioxidants, Cell death/drug effects

## Abstract

**Objective:**

To evaluate the phytochemical composition of hydroethanolic extracts from powdered aerial parts of *Turnera diffusa* Willd (Turneraceae; *T. diffusa*), as well as its toxicity in astrocytes.

**Methods:**

Chemical analyses of hydroethanolic extract from powdered aerial parts of *T. diffusa* were carried out using HPLC-DAD-ESI-MS/MS. *In vitro* assays using astrocytes culture were performed to evaluate cell death.

**Results:**

Flavone-C, O-diglycosides, such as, luteolin-8-C-[6-deoxy-2-O-rhamnosyl]-xylo-hexos-3-uloside, apigenin-8-C-[6-deoxy-2-O-rhamnosyl]-xylo-hexos-3-uloside and apigenin-7-O-6”*-p-*coumaroylglucoside were the main compounds found in this hydroethanolic extract. Concentration time-effect demonstrated the toxicity of this extract at a concentration of 1,000µg/mL in astrocyte culture, after 6 and 24 hours of incubation.

**Conclusion:**

In phytochemical analyses, important antioxidants (mainly flavonoids) were observed. *T. diffusa* extracts presented cytotoxic effect in high concentrations, leading to increased cell death in astrocyte culture.

## INTRODUCTION

The genus *Turnera* (*Turneraceae*) encompasses 135 species from tropical parts of the Americas and Africa.^1^ *Turnera diffusa* Wild ex Schult (*T. diffusa*), known in Brazil as “damiana”, is the most important *Turnera* species, which has many applications in traditional medicine. This species is found in Mexico, Central America, the Caribbean Islands and parts of South America. It has several traditional uses as an aphrodisiac, for hepatic symptoms, depression, anxiety and neurosis, as well as expectorant, stimulant and tonic; being also used to flavor desserts and beverages.^2,3^

Previous works have analyzed *T. diffusa* from a phytochemical perspective. In general, the aerial parts (stems and leaves) of this species show good antioxidant activity, similar to that exhibited by quercetin.^4^ Moreover, alkaloids, cyanogenic glycosides, flavonoids, and volatile oils are the main classes of phytoconstituents found in *Turnera* genus.^2,4-8^

Potential therapeutic uses of *T. diffusa* have been described in previous studies, including gastroprotective, anti-ultra violet radiation and anti-oncogenic effects,^9-12^ all of them depending in some extent of *T. diffusa*’s antioxidant properties. Despite of these potential positive effects, none study evaluated the toxicity threshold of *T. diffusa.* Cell death (which includes apoptosis and necrosis) is closely related to oxidative stress;^13,14^ however, up to now none report has been performed in order to evaluate the effects of *T. diffusa* on cell death.

## OBJECTIVE

To evaluate the phytochemical composition of hydroethanolic extracts from powdered aerial parts of *Turnera diffusa* Willd, as well as its toxicity in astrocytes.

## METHODS

### Plant material

Powdered aerial parts of *T. diffusa* were obtained commercially, from Quimer Ltda and this botanical material was deposited at *Herboteca Carlos Stellfeld* in the *Universidade Federal do Paraná* (UFPR), under number 340.^5^

### Drugs and preparation of extracts

Quercetin, apigenin, kaempferol and luteolin were purchased from Sigma-Aldrich Chemical Co. (Saint Louis, MO, USA). The solutions of these standards (100µg/mL in ethanol) were prepared and analyzed by high-performance liquid chromatography coupled to diode-array detector (HPLC-DAD), in order to carry out the optimization of chromatographic conditions for analysis of hidroethanolic extract. HPLC-grade methanol was purchased from Merck (Darmstadt, Germany). HPLC-grade water was prepared from distilled water using a Milli-Q system (Millipore, Waters, Milford, MA, USA). Powdered aerial parts from *T. diffusa*, (100g) were extracted with 1L of hydroethanolic solution (50%, v/v) by turbolysis, as described by Bezerra et al.^5^ The solutions were filtered, concentrated under reduced pressure in a rotary evaporator, lyophilized and stored in amber flasks at 5°C.

High-performance liquid chromatography coupled to diode-array detector and electrospray ionization - mass spectrometry/mass spectrometry analysis of hydroethanolic extract.

The lyophilized extract (8mg) was dissolved in 3mL of methanol: water (20:80, v/v) and filtered through a 0.45µm filter (German Sciences, Tokyo, Japan). An aliquot 31.2µL of lyophilized extract was injected into the HPLC system. All solvents were of HPLC grade and were filtered using a solvent filtration apparatus. A DAD SPD-M10AVP (Shimadzu) equipped with a photodiode array detector was coupled to an Esquire 3000 Plus mass spectrometer (Bruker Daltonics) with an electrospray ionization (ESI) source and ion trap mass analyzer. Double in-line detection was carried out in the DAD, using 270 and 340nm as preferred wavelengths, and in-line ultraviolet spectrum were recorded in the range of 200 to 400nm. A reverse phase C18 Zorbax - 5B - RP-18 (Hewlett Packard) column (4.6x250mm, 5μm) with a precolumn guard filter was employed for separation of constituents. The mobile phases consisted of A (0.1% aq. formic acid) and B (methanol). The elution gradient was: zero minute – 20% B in A; 10 minutes – 30% B in A; 20 minutes – 50% B in A; 30 minutes – 70% B in A; 40 minutes – 90% B in A; 45 minutes – 40% B in A; and 50 minutes – 20% B in A. The flow rate was kept constant at 0.5mL.min^1^, and the temperature of the column was maintained at 28°C. The mass spectrometry (MS) analysis was carried out using ESI at atmospheric pressure in positive and negative ion modes. The ionization conditions were adjusted as follows: ion source electrospray voltage of -40V, capillary voltage of 4,500V and capillary temperature of 325°C. Helium was used as the collision gas and nitrogen as the nebulizing gas. Nebulization was aided with a coaxial nitrogen sheath gas provided at a pressure of 27psi. Desolvation was assisted using a counter current nitrogen flow set at a flux of 7.0L.min^-1^. Full scan mass acquisitions were performed in both negative and positive ion modes by scanning the m/*z* range from 100 to 1,000 mass units. Collision induced dissociation (CID) spectra were obtained in the ion trap using helium as the collision gas, with voltage ramping cycles from 0.5 to 1.3V. Characterization of constituents was carried out based in UV and mass spectra data, together with fragmentation profiles obtained through mass spectrometry/mass spectrometry (MS/MS spectra), which were compared with literature data and also based on constituents reported for this specie in another studies.

### Culture of astrocytes of rat cortex

A culture of astrocytes was obtained from the cortex of rats 3 days old, as described previously by Smaili and Russell.^15^ The animals were obtained from *Instituto Nacional de Farmacologia* of *Universidade Federal de São Paulo* (UNIFESP). This project was approved by the Ethics Committee of UNIFESP (0464/05).

The cells were grown in low glucose Dulbecco’s modified Eagle’s medium containing 10% fetal bovine serum, 0.1% fungizone, 1% penicillin/streptomycin, 1mM sodium pyruvate and 4mM L-glutamine in 5% CO_2_/95% air. The media was replaced every other day until confluency (after 8 to 14 days). When confluency was reached, the removal of the cells from the flasks was performed by trypsinization. For that, trypsin/EDTA (0.25%) was added to the flasks for 5 minutes, at 37°C, followed by enzyme inactivation by DNAse (10mM) and centrifugation (2,500rpm, 10 minutes, 25°C). Cells were then suspended in culture medium. After trypsinization, 10μL of the suspension were quantified using a Neubauer chamber, an inverse microscope and a cell counter. The total amount of cells was of 72x10^4^ cell/mL. After dilutions, the cells were platted in a 24 wells plate, for a final concentration of 18,000 cells/well, which allowed cells observation and counting.

### Evaluation of cell death using Hoechst 33342

Cell morphology was evaluated by fluorescence microscopy following Hoechst 33342 staining. The hydroethanolic extract from *T. diffusa* was added to the cells at initial concentrations of 10, 100 and 1,000µg/mL and were evaluated after 6, 24 and 48 hours of incubation to assess the toxicity of the extracts. Control cells did not receive any treatment. These cells were also used in other experiments not reported here.

After a separate incubation, cells were washed with buffer and stained with Hoechst 33342 (1µg/mL) for 15 minutes, at room temperature, in the dark. Cell death was identified by some characteristics, such as nuclear condensation, formation of membrane blebs and apoptotic bodies. Fragmented nuclei or nuclei with condensed chromatin were counted and the percentage of these nuclei relative to the total nuclei observed in each field was calculated. The results at each concentration and incubation time were determined from, at least, four independent experiments performed in triplicate.^16^

### Statistical analysis

Data analysis was performed using One-Way Analysis Of Variance (ANOVA) followed by Duncan’s test when appropriate. The results are expressed as the mean ± standard error (SE) of the mean and a significance level of 5% was adopted.

## RESULTS

### Phytochemical analyses

**Figure 1:**
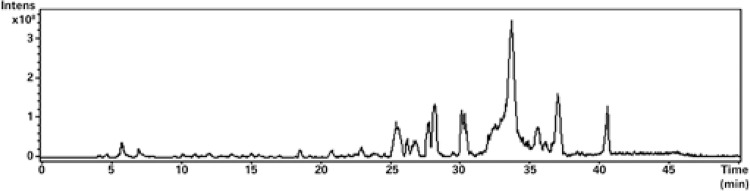
Total ion current chromatogram obtained through electrospray ionization

Table 1 list the retention times (Rt), MS spectral data and wavelength of maximal absorption (λ_max_) for the chemical constituents found in this hydroethanolic extracts. The characterization of constituents was achieved, through MS spectral data reported previously for compounds found in this plant or other plant materials. The total ion current chromatogram obtained in ESI-MS negative ionization mode is shown in figure 1.

**Table 1:** Retention times, mass spectral data and wavelength of maximal absorption (λmax) for the chemical constituents found in hydroethanolic extracts from *Turnera diffusa*

Peak	Rt min	λ_max_ (nm)	(ESI+, *m/z*)	(ESI-, *m/z*)		Proposed structure
	
			[M + H]^+^	[M – H]^-^	MS/MS	
1	23.5	265 and 345	-	593	473 (100), 429 (40), 357 (50) and 327 (80)	Luteolin-6-*C*-glucosyl-2”-O-rhamnoside
2	24.0	265 and 345	-	567	521	Luteolin-8-*C*-(6”-*O*-*p*-benzoyl)-glucoside
3	25.5	260 and 355	-	641	317	Myricetin-3-O-diglucoside
4	26.2	ND	-	743	605 (100)	Luteolin-8-*C*-(*O*-*p*-feruloyl-2”-O-benzoyl) glucoside
5	27.7	260 and 355	-	625	301	Quercetin-3-O-diglucoside
6	28.2	260 and 355	-	655	331	Laricitin-3-O-diglucoside
7	30.1	ND	-	639	575	Quercetin-*O*-caffeoyl glucuronide
8	30.8	252, 265 and 357	-	669	345	Syringetin-3-O-diglucoside
9	33.6	270 and 345	577	575	411 (100), 301 (60) and 285 (20)	Luteolin-8-C-[6-deoxy-2-O-rhamnosyl]-xylo-hexos-3-uloside,
10	35.5	270 and 340	561	559	395 (100), 321 (20) and 269 (20)	Apigenin-8-C-[6-deoxy-2-O-rhamnosyl]-xylo-Hexos-3-uloside,
11	37.0	270 and 315	579	577	269	Apigenin-7-O-(6”O*-p-* coumaroyl)-glucoside

Rt min: minimum retention times; ESI: electrospray ionization; M-H: deprotonated molecule; M+H: protonated molecule; MS: mass spectrometry; ND: not determined.

Mass spectrum analysis in negative ionization mode from hydroethanolic extract from *T. diffusa* is showed in figure 1. Peak number are correspondent to the compounds disclosed at table 1.

The main constituents found in *T. diffusa* were flavonoids, a mixture of flavone-*C, O*-diglycosides, *C*-glycosyl flavone acylated with aromatic acids and flavonols 3-O-diglucosides.

Compounds 1, 2, 9 and 10 exhibited UV spectra characteristic of flavones. The ESI-MS spectrum of compound 9, the main constituent found in this extract, showed deprotonated and protonated molecule at m/*z* 575 and m/z 577, respectively. After MS/MS experiments, fragment ion at m/*z* 411 was abundantly produced, which correspond to the loss of 164 mass units, sugar (rhamnose) and water moieties (146 + 18), characteristic of a bond between a sugar (rhamnose) and a non-phenolic hydroxyl group, most likely at the 2”-*O* position of the other sugar moiety, indicating an *O-*glycosylation with a deoxyhexose (rhamnose). This fragmentation pattern in negative ionization mode was typical of an *O,* C-diglycosylflavone. An unusual fragment ion occurred at m/*z* 301 (60%), together with the fragment ion corresponding to the aglycone luteolin at m/*z* 285 (20%).

Compound 10 showed deprotonated and protonated molecules at m/*z* 559 and m/z 561, respectively, in the ESI-MS spectra. The MS/MS spectrum of the precursor ion at m/*z* 559 showed abundant fragment ion at m/*z* 395, indicating 2”-O-glycosylation with deoxyhexose (rhamnose), and fragment ion at m/*z* 269 (20%), indicating apigenin as aglycone.

The ESI-MS spectrum of compound 2 showed deprotonated molecule at m/*z* 567, which after MS/MS experiments produced fragment ion at m/*z* 521, corresponding to the loss of 46 mass units [HCOOH], which could occur through decarboxylation of a carboxylic acid from benzoic acid moiety. The sugar moiety linked to luteolin could be glucose substituted with benzoic acid on the hydroxyl group in position 6”-O of glucose because, in this position, the cleavage of benzoyl group was more difficult. Compound 2 was tentatively identified as luteolin-8-*C*-(6”-*O*-*p*-benzoyl) glucoside.

The ESI-MS spectrum of compound 4 exhibited deprotonated molecule at m/*z* 743. The molecular mass of compound 4 indicated the presence an additional feruloyl moiety (176 mass units) in its structure, when compared to compound 2. The exact location of the acyl group on the glycosidic moiety was difficult to determine, just based on MS. However, the MS/MS spectrum of the precursor ion at m/*z* 743 showed abundant fragment ion at m/*z* 605, corresponding to the loss of 137 mass units (hydroxybenzoic acid moiety), what could indicated that the hydroxybenzoic acid was attached to glucose at 2”-O-position. Compound 4 was tentatively assigned as luteolin-8-*C*-(*O*-*p*-feruloyl-2”-O-benzoyl)-glucoside.

For compound 1, the ESI-MS spectrum showed deprotonated molecule at m/*z* 593, which after MS/MS experiments produced abundant fragment ion at m/*z* 473, characteristic of the presence of hexose as *C*-glycosylated sugar moiety. The fragment ions at m/*z* 357 (A + 71) and m/*z* 327 (A + 41) were characteristic of flavones-*O*, *C*-diglycosides and suggested luteolin as the aglycone.

In the Rt 25.5 minutes a co-elution of two compounds was observed, compound 3 and another constituent in minor content, that exhibited deprotonated molecular ion at m/z 593 and was tentatively characterized as quercetin dirhamnoside. Compounds 3, 5, 6 and 8 exhibited UV spectra characteristic of flavonols and their ESI-MS spectra exhibited deprotonated molecules at m/z 641, 625, 655 and 669, respectively. The ESI-MS/MS spectra of these compounds in negative ionization mode showed the loss of 324 mass units, indicating the presence of two hexoses (glucoses), probably linked at 3-O-position of aglycone. Thus, compound 3 was suggested as myricetin-3-*O*-diglucoside, compound 5 as quercetin-3-*O*-diglucoside, compound 6 as laricitin-3-*O*-diglucoside and compound 8 as syringetin-3-*O*-diglucoside.

The MS spectrum of compound 7 showed deprotonated molecule at m/*z* 639, and after MS/MS experiments exhibited abundant fragment ion at m/*z* 575, corresponding to the loss of 64 mass units, which could be attributed to a combined loss of COOH and OH groups. According to MS spectral data compound 7 was suggested as quercetin caffeoyl glucuronide.

The UV maximum absorption at 312nm and the higher Rt indicated that compound 11 was a flavonoid glycoside esterified with aromatic acids. The ESI-MS spectra exhibited protonated and deprotonated molecules at m/*z* 579 and 577, respectively. The MS/MS spectrum of the precursor ion at m/*z* 577 showed abundant fragment ion at m/*z* 269, which was attributed to the loss of 308 mass units (glucose with a *p*-coumaroyl moiety), and indicated apigenin as aglycone.

### Evaluation of cell death using Hoechst 33342

**Figure 2:**
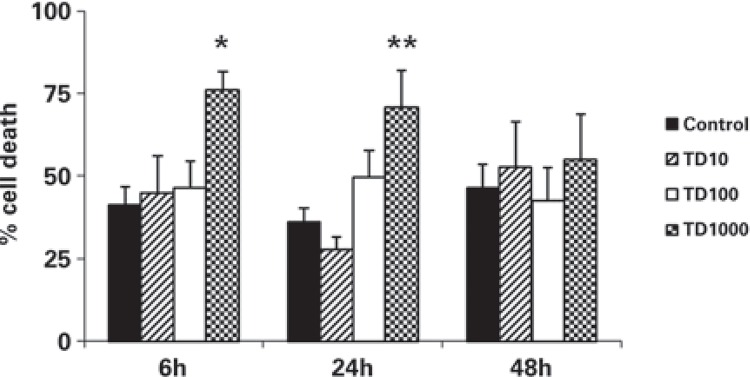
Dead cells after incubation for 6, 24 and 48 hours, at doses of 10, 100 and 1,000µg/mL *Turnera diffusa* extract

**Figure 3:**
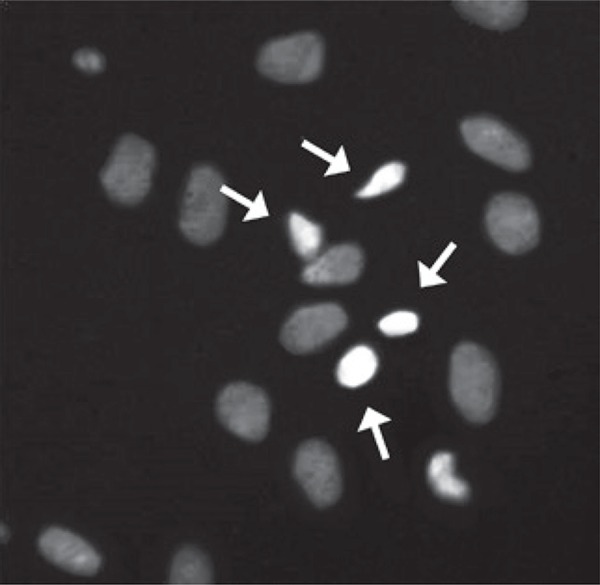
Nuclei of astrocytes marked with Hoechst 33342 and examined under a fluorescence microscope. Arrows identify the nuclei of dead cells and the remaining nuclei represent live cells

ANOVA detected a significant increase on the percentage of dead cells after incubation for 6 hours, with the *T. diffusa* extract, at a concentration of 1,000µg/mL, when compared to the control group and with lower concentrations [F_(3,32)_=3.03; p<0.05] (Figure 2). This effect persisted in cells evaluated after 24 hours of incubation [F_(3,32)_= 5.19; p<0.05], but only when compared with control group and with concentration of 10µg/mL (Duncan; p<0.01). Figure 3 presents an example of Hoechst 33342 staining, showing both live and death astrocyte nuclei.

The data represent the mean ± SEM of four independent experiments done in triplicate. Analysis of Variance/Duncan: *p<0.05 (compared to all other groups, at 6 hours); **p<0.05 (compared to the control group and the group TD10, at 24 hours).

## DISCUSSION

The main constituents found in this extract were flavonoids (flavones and flavonols glycosides). Flavonols and flavones were distinguished by their UV spectra with maxima at 255/370 and 268/340nm, respectively, and also fragmentation pattern of C-glycosyl and O-glycosyl flavonoids. Flavonoids C-glycosides are stable towards acid hydrolysis, due to glycosidic C-C bond and the main fragmentations take place in the sugar, which possess the weakest bonds.^17,18^ Flavonoid *O*-glycosides are bounded to a sugar with formation of an acid labile glycosidic O-C bond. Fragmentation of these flavonoids involves the cleavage at the glycosidic-*O*-linkage with a concomitant H-rearrangement leading to the elimination of the saccharide residue.^18-21^ For flavonols, the 3-OH and 7-OH positions are regular glycosilation sites.^22^

According to MS spectral data reported by Ferreres et al.^23^ and Figueirinha et al.,^24^ compound 9 found in the present study was characterized as luteolin-8-C-[6-deoxy-2-O-rhamnosyl]-xylo-hexos-3-uloside, which was also found by Zhao et al.,^7,8^ in *T. diffusa*, and by Figueirinha et al.,^24^ in *Cymbopogon citratus*. Conforming to MS spectral data reported by Ferreres et al.,^23^ compound 10 was identified as apigenin-8-C-[6-deoxy-2-O-rhamnosyl]-xylo-hexos-3-uloside, which was reported in *Passiflora edulis*.^23^ Arbutin (4-hydroxyphenyl β-D-glucoside) and *p*-coumaroyl glycosides were reported in this species.^7,8^

In consonance to MS data reported by Ferreres et al.,^17,23^ compound 1 was tentatively identified as luteolin-6-*C*-glucosyl-2”-O-rhamnoside. The fragment ion at m/*z* 429 attributed to the loss of rhamnose and water moieties indicated O-glycosylation with a deoxyhexose (rhamnose) at the 2”-*O* position of glucose.^23^

Regarding compounds 3, 5, 6 and 8, Fracassetti et al.^25^ discuss that flavonol-O-glycoside with a free hydroxyl at 3-O position show a UV band I maximum at 374nm, while flavonol with a blocked hydroxyl at 3-O position of aglycone showed UV band I maximum at 356nm. The introduction of methyl groups into the flavonols increases the Rt,^18^ and the elution order was found to be myricetin (m/*z* 317), quercetin (m/*z* 301), laricitin (m/*z* 331) and syringetin (m/*z* 345). Laricitin-3-*O*-diglucoside and syringetin-3-*O*-diglucoside were reported in *T. diffusa* by Zhao et al.^7,8^ and Szewczyk and Zidorn.^2^

Generally, flavonoid glycosides esterified with aromatic acids have longer Rt on RP-HPLC columns than diglycosides and monoglycosides and its UV spectra exhibit an intense band I (approximately 330nm) and a small band II at 270nm, resulting from the UV absorption overlap of the flavonoid and the cinnamoyl acid.^18^ The predominant site of bonding of the acyl groups is usually the 6”-position of hexose, although other positions should not be excluded.^18^ Compound 11 was identified as apigenin-7-*O*-(6”-O-*p*-coumaroyl) glucoside, which was found in high concentration in this species by Camargo and Vilegas.^26^

Some studies had shown that flavonoids from diverse herbal medicines have significant effects in different developmental stages of nervous systems, including neuronal stem cell differentiation, neurite outgrowth, and neuronal plasticity.^27,28^ Polyphenols and flavonoids present in *Ocimum sanctum* hydroalcoholic extract ameliorated H_2_O_2_ induced neuronal damage through its antioxidant defense mechanism, exhibiting potential to be used to treat oxidative stress mediated neuronal disorders.^29^ The neuroprotective actions of flavonoids are believed to occur through direct interactions with cellular cascades, yielding expression of neuroprotective and neuromodulatory proteins that promote neurogenesis, neuronal function and brain connectivity, and also through blood-flow improvement and angiogenesis in the brain and sensory systems.^30^

Previous studies demonstrated neuroprotective effects by luteolin, through increasing the neuronal viability, reducing the number of apoptotic cells^31^ and reducing ischemia-induced cellular injury.^32^ The prevention of oxidative stress, had been attributed to flavonoids, which can also modulate both enzymes and receptors activities, acting as multi-target botanical therapeutics or drugs.^33^ However, in certain concentrations, flavonoids can also act as pro-oxidants, depleting the nuclear antioxidant defense systems and leading to oxidative DNA damage.^34^ This finding is in accordance to our results that showed that high concentrations of *T. diffusa* could cause cell death in astrocytes.

The *T. diffusa* extract demonstrated toxicity at a concentration of 1,000µg/mL after both 6 and 24 hours of incubation. However, concentrations of 10 and 100µg/mL of extract showed similar effects as those observed in the control group, which underscores the low cellular toxicity of these doses during this period. Thus, one may note that *T. diffusa* has a dose-dependent toxicity. The toxicity threshold for this plant lies between 100 and 1,000µg/mL, for astrocyte death. This dose-dependent relationship and this estimative for a toxicity threshold may explain, to a certain extent, the opposition in anti- and pro-oxidative effects.

It was expected that after 48 hours of incubation, 1,000µg/mL concentrations of the *T. diffusa* extract would also decrease the viability of astrocytes, but the results indicated that the cells did not differ from the control. This result may have occurred because the control cells began to run out of nutrients after 48 hours in unchanged medium, leading to a loss of adherence and natural death.

For the proper assessment of these results, some limitations must be taken into account. First, one must bear in mind that, despite being a widely used method, Hoechst 33342 is a highly unspecific technique. Thus, it is not possible to determine which is the real type or source of the cell death observed in these experiments. In other words, it is not possible to determine if cell death was caused by apoptosis, necrosis or some other phenomena. Another important caveat relies on the establishment of a toxicity threshold. In accordance to our experiments, it is possible to affirm that the toxicity threshold lies somewhere between 100 and 1,000µg/mL. This is a quite large interval, and the lack of other experiments evaluating the toxic effects of *T. diffusa* prevents the establishment of a more precise toxicity level. Moreover, one must bear in mind that this toxicity level applies exclusively for cell death observed in astrocytes. Any other type of toxic effect, as well as effects in other types of cells and tissues, may have their own toxic levels. On the other hand, it can be assumed that further studies on potential therapeutic effects of *T. diffusa* may focus on doses lower than 100µg/mL. As final caveat relies the phytochemical profile observed in the current experiment. As in great part of the experiments carried out with medicinal plants, the extracted compounds depend upon environmental factors (planting site, soil characteristics, temperature and humidity, among others), harvest and post-harvest procedures (drying, milling and storage) and extraction methods (different techniques, solvents and concentrations). Thus, the acquired results should be contextualized to the characteristics of this experiment. It is highly likely that other experiments using *T. diffusa* extract present similar phytochemical profile, but some variations are expected, reason why phytochemical analysis are always useful in this kind of experiments.

## CONCLUSION

*Turnera diffusa* Willd (Turneraceae) is used by Brazilian population and the evaluation of its efficacy have value under ethnopharmacological point of view. Chemical analysis revealed the presence of flavone-C, O-diglycosides, such as luteolin-8-C-[6-deoxy-2-O-rhamnosyl]-xylo-hexos-3-uloside, apigenin-8-C-[6-deoxy-2-O-rhamnosyl]-xylo-hexos-3-uloside and apigenin-7-O-6”-*p-*coumaroylglucoside in high concentrations in this hydroethanolic extract. Hydroethanolic extract of *Turnera diffusa* presented cytotoxic activity and increased the percentage of astrocyte death only at 1,000µg/mL, both on the conditions of 6 and 24 hours of exposure.
