# Supplementary material for: Phytochemical analysis of hydroethanolic extract of Turnera diffusa Willd and evaluation of its effects on astrocyte cell death
Source: Einstein (Sao Paulo). 2016 Jan-Mar;14(1):56–63. doi: 10.1590/S1679-45082016AO3386 (PMC4872919; doi:10.1590/S1679-45082016AO3386)
Supplement: Translation

# Translation: Análise fitoquímica do extrato hidroetanólico de *Turnera diffusa* Willd e avaliação de seus efeitos na morte de astrócitos

**Authors:** Andréia Gomes Bezerra, Giuseppina Negri, Joaquim Maurício Duarte-Almeida, Soraya Soubhi Smaili, Elisaldo Araújo Carlini

**Affiliations:** 1 Universidade Federal de São Paulo, São Paulo, SP, Brasil.; 2 Universidade Federal de São João del-Rei, Divinópolis, MG, Brasil.

**Keywords:** Cognição/efeito de drogas, Extratos vegetais/toxicidade, Substâncias protetoras/toxicidade, Antioxidantes, Morte celular/efeitos de drogas

## Abstract

**Objetivo:**

Avaliar a composição fitoquímica do extrato hidroetanólico das partes aéreas de *Turnera diffusa* Willd (Turneraceae; *T. diffusa*) e sua toxicidade em astrócitos.

**Métodos:**

Análises químicas do extrato hidroetanólico de partes aéreas de *T. diffusa* foram feitas por HPLC-DAD-ESI-MS/MS. Os ensaios *in vitro* utilizaram culturas de astrócitos para avaliar morte celular.

**Resultados:**

Flavonas-C, O-diglicosídeos, como, luteolina-8-C-[6-deoxi-2-O-raminosil]-xilo-hexos-3-ulosideo, apigenina-8-C-[6-deoxi-2-O-raminosil]-xilo-hexos-3-ulosideo e apigenina-7-O-6”-*p-*cumaroilglucosídeo foram os principais constituintes encontrados neste extrato hidroetanólico. Uma curva tempo-concentração demonstrou toxicidade desse extrato na concentração de 1.000µg/mL, na cultura de astrócitos após 6 e 24 horas de incubação.

**Conclusão:**

Nas análises fitoquímicas, importantes antioxidantes, sobretudo flavonoides, foram observados. Extratos de *T. diffusa* apresentaram efeitos citotóxicos em altas concentrações, ocasionando aumento de morte celular em cultura de astrócitos.

## INTRODUÇÃO

O gênero *Turnera* (*Turneraceae*) engloba 135 espécies de áreas tropicais das Américas e da África.^1^ *Turnera diffusa* Willd ex Schult (*T. diffusa*), conhecida no Brasil como “damiana”, é a espécie de *Turnera* mais importante, muito utilizada na medicina tradicional. É encontrada no México, América Central, ilhas do Caribe e certas áreas da América do Sul. Tradicionalmente é utilizada como afrodisíaco, para alívio de sintomas hepáticos, depressão, ansiedade e neurose, e também como expectorante, estimulante e tônico. É ainda empregada para dar sabor a sobremesas e bebidas.^2,3^

Estudos anteriores analisaram a *T. diffusa* a partir da perspectiva fitoquímica. Em geral, os componentes aéreos (caules e folhas) da espécie apresentam boa atividade antioxidante, semelhante à da quercetina.^4^ Além disso, os alcaloides, glicosídeos cianogênicos, flavonoides, e óleos voláteis são as principais classes de fitoconstituintes encontradas no gênero *Turnera.* ^2,4-8^

As possíveis aplicações terapêuticas da *T. diffusa* foram descritas em estudos anteriores, incluindo os efeitos gastroprotetores, antirradiação ultravioleta e antioncogênicos,^9-12^ todos eles dependendo, até certo ponto, das propriedades antioxidantes da *T. diffusa.* Apesar desses possíveis efeitos positivos, nenhum estudo avaliou o limiar de toxicidade da *T. diffusa.* A morte celular (que inclui apoptose e necrose) está estreitamente relacionada ao estresse oxidativo;^13,14^ mas, até agora, nenhuma publicação avaliou os efeitos da *T. diffusa* sobre a morte celular.

## OBJETIVO

Avaliar a composição fitoquímica do extrato hidroetanólico das partes aéreas de *Turnera diffusa* Willd e sua toxicidade em astrócitos.

## MÉTODOS

### Material vegetal

Partes aéreas em pó de *T. diffusa* foram adquiridas comercialmente da Quimer Ltda. Esse material botânico foi depositado na Herboteca Carlos Stellfeld, da Universidade Federal do Paraná (UFPR), sob número 340.^5^

### Fármacos e preparação de extratos

Quercetina, apigenina, campferol e luteolina foram adquiridos da Sigma-Aldrich Chemical Co. (Saint Louis, MO, EUA). As soluções desses padrões (100µg/mL em etanol) foram preparadas e analisadas por cromatografia líquida de alta eficiência acoplada a um detector de arranjo de diodos (HPLC-DAD - *high-performance liquid chromatography coupled to diode-array detector*), a fim de otimizar as condições cromatográficas para análise do extrato hidroetanólico. Metanol grau HPLC foi adquirido da Merck (Darmstadt, Alemanha). Água grau HPLC foi obtida a partir de água destilada usando sistema Milli-Q (Millipore, Waters, Milford, MA, EUA). Partes aéreas em pó de *T. diffusa* (100g) foram extraídas com 1L de solução hidroetanólica (50%, v/v) por turbólise, conforme descrito por Bezerra et al.^5^ As soluções foram filtradas, concentradas sob pressão reduzida em um evaporador rotativo, liofilizadas e armazenadas em frascos âmbar, a 5°C.

Análise do extrato hidroetanólico por cromatografia líquida de alta eficiência acoplada com um detector de arranjo de diodos e espectrometria de massa com ionização por eletrospray/espectrometria de massa.

O extrato liofilizado (8mg) foi dissolvido em 3mL de metanol: água (20:80, v/v) e filtrado em filtro de 0,45µm (German Sciences, Tóquio, Japão). Uma alíquota de 31,2µL de extrato liofilizado foi injetada no sistema HPLC. Todos os solventes utilizados eram grau HPLC e foram filtrados por um aparelho de filtragem de solventes. Um DAD SPD-M10AVP (Shimadzu) equipado com um detector de arranjo de fotodiodos foi acoplado a um espectrômetro de massa Esquire 3000 Plus (Bruker Daltonics) com uma fonte de ionização por eletrospray (ESI - *electrospray ionization*) e analisador de massa para armadilha de íons (*ion-trap*). Dupla detecção em linha foi realizada no DAD, dando-se preferência aos comprimentos de onda de 270 e 340nm, e o espectro ultravioleta em linha foi registrado no intervalo de 200 a 400nm. Uma coluna de fase reversa C18 Zorbax - 5B - RP-18 (Hewlett Packard; 4,6x250mm, 5μm) com filtro pré-coluna de guarda foi empregada para a separação dos componentes. As fases móveis consistiram de A (0,1% aq. de ácido fórmico) e B (metanol). O gradiente de eluição foi: zero minuto – 20% B em A; 10 minutos – 30% B em A; 20 minutos – 50% B em A; 30 minutos – 70% B em A; 40 minutos – 90% B em A; 45 minutos – 40% B em A; e 50 minutos – 20% B em A. A vazão foi mantida constante a 0,5mL.min^-1^, e a temperatura da coluna foi mantida a 28°C. A análise de espectrometria de massa (MS - *mass spectrometry*) foi realizada utilizando ESI sob pressão atmosférica, nos modos de íons positivos e negativos. As condições de ionização foram ajustadas da seguinte forma: eletrospray de fonte iônica tensão de -40V, voltagem capilar de 4.500V e temperatura capilar de 325°C. Hélio foi utilizado como gás de colisão, e nitrogênio como de nebulização. A nebulização foi auxiliada por gás de bainha nitrogênio coaxial à pressão de 27psi. A dessolvatação foi auxiliada pelo uso de vazão de nitrogênio em contracorrente ajustada para um fluxo de 7,0L.min^-1^. Aquisições em massa de varredura completa foram realizadas tanto no modo de íons positivos quanto negativos, por varredura do intervalo m/*z* de 100 a 1.000 unidades de massa. Os espectros de dissociação induzida por colisão (CID - *collision induced dissociation*) foram obtidos na armadilha de íons utilizando hélio como gás de colisão, com ciclos de rampa de tensão de 0,5 a 1,3V. A caracterização dos componentes foi feita com base em UV e dados de MS, juntamente de perfis de fragmentação obtidos de espectrometria de massa/espectrometria de massa (espectros MS/MS), que foram comparados aos dados da literatura, e também com base em componentes relatados para essa espécie, em outros estudos.

### Cultura de astrócitos de córtex de ratos

Uma cultura de astrócitos foi obtida a partir do córtex de ratos de 3 dias de idade, conforme descrito anteriormente por Smaili e Russell.^15^ Os animais foram obtidos do Instituto Nacional de Farmacologia da Universidade Federal de São Paulo (UNIFESP). Este projeto foi aprovado pelo Comitê de Ética da UNIFESP (0464/05).

As células foram cultivadas em um meio Eagle modificado por Dulbecco com baixo teor de glicose, contendo 10% de soro fetal bovino, 0,1% de fungizona, 1% de penicilina/estreptomicina, 1mM de piruvato de sódio e 4mM de L-glutamina em 5% de CO_2_/95% de ar. O meio foi substituído a cada 2 dias, até a confluência (após 8 a 14 dias). Atingida a confluência, a remoção das células dos frascos foi realizada por tripsinização. Para tanto, a tripsina/EDTA (0,25%) foi adicionada aos frascos por 5 minutos, a 37°C, com subsequente inativação da enzima por DNAse (10mM) e centrifugação (2.500rpm, 10 minutos, 25°C). As células foram, então, suspensas no meio de cultura. Após a tripsinização, 10μL da suspensão foram quantificados por meio de uma câmara de Neubauer, um microscópio inverso e um contador de células. A quantidade total de células foi de 72x10^4^ células/mL. Após as diluições, as células foram incubadas em placa de 24 poços, chegando à concentração final de 18.000 células/poço, o que permitiu a observação e a contagem das células.

### Avaliação da morte celular por meio de Hoechst 33342

A morfologia celular foi avaliada por microscopia de fluorescência após coloração com Hoechst 33342. O extrato hidroetanólico da *T. diffusa* foi adicionado às células em concentrações iniciais de 10, 100 e 1.000µg/mL, e avaliado após 6, 24 e 48 horas de incubação, para verificar a toxicidade dos extratos. As células de controle não receberam nenhum tratamento. Essas células também foram utilizadas em outros experimentos não relatados aqui.

Após período de incubação, as células foram lavadas com tampão e coradas com Hoechst 33342 (1µg/mL) durante 15 minutos, à temperatura ambiente, no escuro. A morte celular foi identificada por algumas características, como condensação nuclear, formação de bolhas na membrana e corpos apoptóticos. Os núcleos fragmentados ou com cromatina condensada foram contados e a porcentagem desses núcleos, em relação ao total de núcleos, foi calculada por campo observado. Os resultados para cada concentração e tempo de incubação foram determinados a partir de, pelo menos, quatro experimentos independentes realizados de forma triplicada.^16^

### Análise estatística

A análise dos dados foi realizada utilizando-se o teste de Análise de Variância Univariada (ANOVA), seguido do teste de Duncan, quando apropriado. Os resultados foram expressos como média ± erro padrão (EP) da média e adotaram nível de significância de 5% (p<0,05).

## RESULTADOS

### Análises fitoquímicas

A tabela 1 apresenta os tempos de retenção (Rt - *retention times*), dados espectrais de MS e comprimento de onda de absorção máxima (λ_max_) para os componentes químicos encontrados nos extratos hidroetanólicos. A caracterização dos componentes foi alcançada por meio de dados espectrais de MS reportados anteriormente para os compostos relacionados a este ou outros materiais vegetais. O cromatograma de corrente iônica total obtido no modo de ionização negativa do ESI-MS está representado na figura 1.

**Tabela 1:** Tempos de retenção, dados de espectro de massas e o comprimento de onda de absorção máxima (λmax) para os componentes químicos encontrados em extratos hidroetanólicos de *Turnera diffusa*

Pico	Rt min	λ_max_ (nm)	(ESI+, *m/z*)	(ESI-, *m/z*)		Estrutura proposta
			[M + H]^+^	[M – H]^-^	MS/MS	
1	23,5	265 e 345	-	593	473 (100), 429 (40), 357 (50) e 327 (80)	Luteolina-6-*C*-glucosil-2”-O-ramnosídeo
2	24,0	265 e 345	-	567	521	Luteolina-8-*C*-(6”-*O*-*p*-benzoila)-glucosídeo
3	25,5	260 e 355	-	641	317	Miricetina-3-O-diglucosídeo
4	26,2	ND	-	743	605 (100)	Luteolina-8-*C*-(*O*-*p*-feruloil-2”-O-benzoil)-glucosídeo
5	27,7	260 e 355	-	625	301	Quercetina-3-O-diglucosídeo
6	28,2	260 e 355	-	655	331	Laricitina-3-O-diglucosídeo
7	30,1	ND	-	639	575	Quercetina-*O*-cafeoilglucuronida
8	30,8	252, 265 e 357	-	669	345	Siringetina-3-O-diglucosídeo
9	33,6	270 e 345	577	575	411 (100), 301 (60) e 285 (20)	Luteolina-8-C-[6-desoxi-2-O-ramnosil]-xilo-hexos-3-ulosideo
10	35,5	270 e 340	561	559	395 (100), 321 (20) e 269 (20)	Apigenina-8-C-[6-desoxi-2-O-ramnosil]-xilo-hexos-3-ulosideo
11	37,0	270 e 315	579	577	269	Apigenina-7-O-(6”O*-p-* cumaroil)-glucosídeo

Rt min: tempo de retenção mínimo; ESI: electrospray ionization; M-H: molécula deprotonada; M+H: molécula protonada; MS: mass spectrometry; ND: não determinado.

**Figura 1:** Cromatograma de corrente de íon total obtido de ionização por eletrospray

A análise de espectrofotometria de massa em modo de ionização negativa do extrato hidroetanólico da *T. diffusa* está representado na figura 1. Os números de pico são correspondentes aos compostos relacionados na tabela 1.

Os principais componentes encontrados na *T. diffusa* foram flavonoides, uma mistura de flavona-*C, O*-diglicosídeos, *C*-glicosil flavona acilada com ácidos aromáticos e flavonóis 3-O-diglucosídeos.

Os compostos 1, 2, 9 e 10 apresentaram espectros de UV característicos de flavonas. O espectro ESI-MS do composto 9, o principal componente encontrado neste extrato, apresentou molécula desprotonada e protonada a m/z 575 e m/z 577, respectivamente. Após experimentos MS/MS, houve produção abundante de íon fragmento a m/*z* 411, que correspondeu à perda de 164 unidades de massa, açúcar (ramnose) e frações de água (146 + 18), característica de uma ligação entre um açúcar (ramnose) e um grupo de hidroxila não fenólica, mais provavelmente na posição 2”-*O* da outra fração de açúcar, indicando glicosilação de *O-* com desoxihexose (ramnose). Esse padrão de fragmentação no modo de ionização negativa é típico de *O*, C-diglicosilflavona. Um íon fragmento incomum ocorreu em m/z 301 (60%), em conjunto com o íon fragmento correspondente à luteolina aglicona a m/*z* 285 (20%).

O composto 10 apresentou moléculas desprotonadas e protonadas a m/*z* 559 e m/z 561, respectivamente, nos espectros de ESI-MS. O espectro MS/MS do íon precursor em m/*z* 559 apresentou abundantes íons fragmentos a m/*z* 395, indicando 2”-O-glicosilação com desoxihexose (ramnose), e íon fragmento a m/*z* 269 (20%), indicando apigenina como aglicona.

O espectro de ESI-MS do composto 2 apresentou molécula desprotonada a m/*z* 567, que, após experimentos MS/MS, produziu íon fragmento a m/*z* 521, correspondente a uma perda de 46 unidades de massa [HCOOH], o que poderia ocorrer por meio de descarboxilação de um ácido carboxílico da fração de ácido benzoico. A fração de açúcar ligada à luteolina poderia ser glicose substituída por ácido benzoico no grupo hidroxila na posição 6 “-O da glicose, porque, nessa posição, a clivagem do grupo benzoíla era mais difícil. O composto 2 foi temporariamente identificado como luteolina-8-*C*-(6”-*O*-*p*-benzoíla) glucosídeo.

O espectro de ESI-MS do composto 4 apresentou molécula desprotonada a m/z 743. A massa molecular do composto 4 indicou presença de uma porção adicional de fração de feruloil (176 unidades de massa) em sua estrutura, quando comparado ao composto 2. A localização exata do grupo acilo na fração glicosídica encontrou dificuldade de ser determinado apenas com base em MS. No entanto, o espectro MS/MS do íon precursor em m/z 743 apresentou abundante íon fragmento a m/*z*605, correspondente à perda de 137 unidades de massa (fração de ácido hidroxibenzoico), o que possibilitou indicar que o ácido hidroxibenzoico estava ligado à glicose na posição 2”-O-. O composto 4 foi provisoriamente identificado como luteolina-8-*C*-(*O*-*p*-feruloil- 2”-O-benzoil)-glucosídeo.

Para o composto 1, o espectro de ESI-MS apresentou molécula desprotonada a m/*z* 593, que, após experimentos MS/MS, produziu abundante íon fragmento a m/*z* 473, característica da presença de hexose como fração de açúcar *C*-glicosilado. Os íons fragmentos a m/*z* 357 (A + 71) e m/*z* 327 (A + 41) eram característicos de flavonas- *O*, *C*-diglicosídeos e sugeriram luteolina como aglicona.

No Rt de 25,5 minutos, foi observada uma co-eluição dos dois compostos, o composto 3 e outro componente em menor teor, que apresentou íon molecular desprotonado a m/z 593 e foi temporariamente caracterizado como diramnosídeo de quercetina. Os compostos 3, 5, 6 e 8 apresentaram espectros UV característicos de flavonóis, e seus espectros de ESI-MS apresentaram moléculas desprotonadas a m/*z* 641, 625, 655 e 669, respectivamente. Os espectros de ESI-MS/MS desses compostos, no modo de ionização negativa, apresentaram perda de 324 unidades de massa, indicando a presença de duas hexoses (glicoses), provavelmente ligadas à posição 3-O- de aglicona. Assim, o composto 3 foi sugerido como miricetina-3-*O*-diglucosídeo, o composto 5 como quercetina-3-*O* -diglucosídeo, o composto 6 como laricitina-3-*O*-diglucosídeo, e o 8 como siringetina-3-*O*-diglucosídeo.

O espectro de MS do composto 7 apresentou molécula desprotonada a m/*z* 639 e, após experimentos de MS/MS, apresentou um abundante íon fragmento a m/*z* 575, correspondente à perda de 64 unidades de massa, o que poderia ser atribuído a uma perda combinada dos grupos COOH e OH. De acordo com os dados espectrais de MS, sugeriu-se que o composto 7 era quercetina cafeoil glucuronida.

A absorção máxima de UV a 312nm e o Rt mais elevado indicaram que o composto 11 era um glicosídeo flavonoide esterificado com ácidos aromáticos. Os espectros de ESI-MS apresentaram moléculas protonadas e desprotonadas a m/*z* 579 e 577, respectivamente. O espectro MS/MS do íon precursor a m/*z* 577 apresentou abundante íon fragmento a m/*z* 269, o que foi atribuído à perda de 308 unidades de massa (glicose com fração *p*-cumaroila) e indicou apigenina como aglicona.

### Avaliação da morte celular por meio de Hoechst 33342

A ANOVA detectou um aumento significativo na porcentagem de células mortas após incubação, por 6 horas, com extrato de *T. difusa*, à concentração de 1.000µg/L, quando comparado com o grupo de controle e a concentrações mais baixas [F_(3,32)_=3,03; p<0,05] (Figura 2). Esse efeito persistiu em células avaliadas após 24 horas de incubação [F_(3,32)_=5,19; p<0,05], mas apenas quando comparado com o grupo de controle e à concentração de 10µg/mL (Duncan; p<0,01). A figura 3 apresenta um exemplo de coloração com Hoechst 33342, exibindo núcleos de astrócitos tanto vivos quanto mortos.

**Figura 2:** Células mortas após incubação por 6, 24 e 48 horas, em doses de 10, 100 e 1.000µg/mL de extrato *Turnera diffusa*

Os dados representam média ± EPM de quatro experimentos independentes realizados em triplicata. Análise de Variância/Duncan: *p<0,05 (comparado com todos os outros grupos após 6 horas); **p<0,05 (comparado com o grupo controle e com o grupo TD10, após 24 horas).

**Figura 3:** Núcleos de astrócitos marcados com Hoechst 33342 e examinados com microscópio de fluorescência. As setas identificam os núcleos de células mortas, e os núcleos restantes representam células vivas

## DISCUSSÃO

Os principais componentes encontrados neste extrato foram flavonoides (flavonas e flavonóis glicosídeos). Flavonóis e flavonas foram diferenciados por seus espectros UV com máximos de 255/370 e 268/340nm, respectivamente, e também pelo padrão de fragmentação de flavonoides C-glicosil e O-glicosil. Os flavonoides C-glicosídeos são estáveis à hidrólise ácida, devido à ligação C-C glicosídica, e as principais fragmentações acontecem no açúcar, que possui as ligações mais fracas.^17,18^ Flavonoides *O*-glicosídeos se ligam a um açúcar com formação de uma ligação glicosídica O-C ácido lábil. A fragmentação desses flavonoides envolve clivagem na ligação *O*-glicosídica, com um concomitante rearranjo-H, que leva à eliminação do resíduo de sacarídeo.^18-21^ No caso dos flavonóis, as posições 3-OH e 7-OH são locais de glicosilação regular.^22^

De acordo com dados espectrais de MS relatados por Ferreres et al.^23^ e Figueirinha et al.,^24^ o composto 9, encontrado no presente estudo, foi caracterizado como luteolina-8-C-[6-desoxi-2-O-ramnosil]-xilo- hexos-3-ulosideo, que também foi encontrado por Zhao et al.^7,8^ na *T. diffusa*, e por Figueirinha et al.^24^, em *Cymbopogon citratus.* Em conformidade com dados espectrais de MS relatados por Ferreres et al.,^23^ o composto 10 foi identificado como apigenina-8-C- [6-desoxi-2-O-ramnosil]-xilo-hexos-3-ulosideo, que foi relatado na *Passiflora edulis.* ^23^ Arbutina (4-hidroxifenil β-D-glucosídeo) e glucosídeos *p*-cumaroil foram relatados nesta espécie.^7,8^

Em consonância com os dados de MS relatados por Ferreres et al.,^17,23^ o composto 1 foi temporariamente identificado como luteolina-6-*C*-glicosil-2”-O-ramnosídeo. O íon fragmento em m/*z* 429 atribuído à perda de ramnose e frações hídricas indicaram O-glicosilação com uma desoxihexose (ramnose) na posição 2”-*O* da glicose.^23^

Em relação aos compostos 3, 5, 6 e 8, Fracassetti et al.^25)^ argumentam que um flavonol-O-glicosídeo com uma hidroxila livre na posição 3-O apresentou uma banda I UV máxima de 374nm, enquanto um flavonol com uma hidroxila bloqueada na posição 3-O da aglicona apresentou banda I UV máxima de 356nm. A introdução de grupos de metila nos flavonóis aumenta o Rt,^18^ e foi determinado que a ordem de eluição seria miricetina (m/*z* 317), quercetina (m/*z* 301), laricitina (m/*z* 331) e siringetina (m/*z* 345). Laricitina-3-*O*-diglucosídeo e siringetina-3-*O*-diglucosídeo foram relatados na *T. diffusa* por Zhao et al.^7,8^ e Szewczyk e Zidorn.^2^

Em geral, os flavonóis glicosídeos esterificados com ácidos aromáticos têm Rt mais longo em colunas RP-HPLC que os diglicosídeos e monoglicosídeos, e seus espectros UV apresentam uma banda I intensa (aproximadamente 330nm) e uma pequena banda II a 270nm, resultante da sobreposição da absorção de UV do flavonoide e do ácido cinamoil.^18^ O local predominante da ligação dos grupos acila é geralmente a posição 6”- da hexose, embora outras posições também devam ser consideradas.^18^ O composto 11 foi identificado como apigenina-7-*O*-(6”-O-*p*-cumaroil)-glucosídeo, encontrado em alta concentração nessa espécie por Camargo e Vilegas.^26^

Alguns estudos mostraram que os flavonoides de diversos fitoterápicos têm efeitos significativos em diferentes estágios de desenvolvimento do sistema nervoso, incluindo a diferenciação neuronal de células-tronco, crescimento de neurites, e plasticidade neuronal.^27,28^ Polifenois e flavonoides presentes no extrato hidroalcoólico de *Ocimum sanctum* melhoraram os danos neuronais induzidos por H_2_O_2_, por meio de seu mecanismo de defesa antioxidante, demonstrando potencial para ser utilizado no tratamento de distúrbios neuronais induzidos pelo estresse oxidativo.^29^ Acredita-se que as ações neuroprotetoras dos flavonoides ocorram por meio de interações diretas com cascatas celulares, gerando expressão de proteínas neuroprotetoras e neuromoduladoras, que promovem a neurogênese, a função neuronal e a conectividade cerebral, e também pela melhora do fluxo sanguíneo e da angiogênese no cérebro e nos sistemas sensoriais.^30^

Estudos anteriores demonstraram os efeitos neuroprotetores da luteolina, por meio do aumento da viabilidade neuronal, da redução do número de células apoptóticas^31^ e da redução da lesão celular induzida por isquemia.^32^ A prevenção do estresse oxidativo foi atribuída aos flavonoides, que também podem modular a atividade tanto de enzimas quanto de receptores, atuando como fitoterápicos ou medicamentos multialvo.^33^ No entanto, em certas concentrações, os flavonoides podem também atuar como pró-oxidantes, esgotando os sistemas de defesa nucleares antioxidantes e levando a danos oxidativos no DNA.^34^ Este achado está de acordo com nossos resultados, que indicaram que altas concentrações de *T. diffusa* poderiam causar morte celular em astrócitos.

O extrato de *T. diffusa* apresentou toxicidade na concentração de 1.000µg/mL depois de 6 e 24 horas de incubação. No entanto, concentrações de 10 e 100µg/mL do extrato apresentaram efeitos semelhantes aos observados no grupo de controle, o que reforça a baixa toxicidade celular dessas doses durante esse período. Assim, pode-se observar que a *T. diffusa* apresenta toxicidade dose-dependente. O limiar de toxicidade para essa planta situa-se entre 100 e 1.000µg/mL para morte de astrócitos. Essa relação dose-dependente e essa estimativa do limiar de toxicidade podem explicar, até certo ponto, a oposição nos efeitos anti e pró-oxidativos.

Esperava-se que, após 48 horas de incubação, concentrações de 1.000µg/mL do extrato da *T. diffusa* também diminuiriam a viabilidade de astrócitos, mas os resultados indicaram que as células não diferiram daquelas do grupo controle. Esse resultado pode ter ocorrido porque os nutrientes das células do controle começaram a se esgotar após 48 horas em meio inalterado, levando a perda de aderência e morte natural.

Para uma avaliação adequada destes resultados, algumas limitações devem ser levadas em conta. Em primeiro lugar, deve-se ter em mente que, apesar de ser um método amplamente utilizado, a Hoechst 33342 é uma técnica altamente inespecífica. Assim, não é possível determinar o verdadeiro tipo de natureza de morte celular observada nestas experiências. Em outras palavras, não é possível determinar se a morte celular foi provocada por apoptose, necrose ou outros fenômenos. Outra ressalva importante deve ser feita em relação à definição do limite de toxicidade. De acordo com nossos experimentos, é possível afirmar que o limiar de toxicidade encontra-se em algum ponto entre 100 e 1.000µg/mL. Este intervalo é bastante amplo, e a falta de outros ensaios que avaliem os efeitos tóxicos da *T. diffusa* impedem o estabelecimento de um nível de toxicidade mais preciso. Além disso, deve-se levar em consideração que esse nível de toxicidade aplica-se exclusivamente à morte celular observada nos astrócitos. Qualquer outro tipo de efeito tóxico, bem como efeitos sobre outros tipos de células e tecidos, pode ter seus próprios níveis de toxicidade. Por outro lado, pode-se supor que futuros estudos sobre os possíveis efeitos terapêuticos da *T. diffusa* se restrinjam a doses inferiores a 100µg/mL. Uma advertência final refere-se ao perfil fitoquímico observado neste experimento. Como acontece em grande parte dos experimentos realizados com plantas medicinais, os compostos extraídos dependem de fatores ambientais (local de plantio, características do solo, temperatura e umidade, entre outros), procedimentos de colheita e pós-colheita (secagem, moagem e armazenamento), e extração (diferentes técnicas, solventes e concentrações). Assim, os resultados obtidos devem ser contextualizados de acordo com as características deste experimento. É altamente provável que outros estudos que utilizem o extrato de *T. diffusa* apresentem perfil fitoquímico semelhante, porém com algumas variações, razão pela qual a análise fitoquímica é sempre útil para esse tipo de ensaio.

## CONCLUSÃO

A *Turnera diffusa* Willd (Turneraceae) é utilizada pela população brasileira, e a avaliação de sua eficácia tem valor do ponto de vista etnofarmacológico. A análise química revelou a presença de flavona-C, O-diglicosídeos, tais como luteolina-8-C-[6-desoxi -2-O-ramnosil]-xilo-hexos-3-ulosideo, apigenina-8-C [6- desoxi-2-O-ramnosil]-xilo-hexos-3-uloside e apigenina-7-O-6”-*p-*cumaroilglucosídeo em altas concentrações neste extrato hidroetanólico. Esses mesmos compostos também foram relatados nessa espécie. O extrato hidroetanólico da *Turnera diffusa* apresentou atividade citotóxica e aumentou a porcentagem de morte dos astrócitos somente em 1.000µg/mL, tanto após 6 quanto após 24 horas de exposição.
